# Long noncoding RNA SNHG6 promotes proliferation and angiogenesis of cholangiocarcinoma cells through sponging miR-101-3p and activation of E2F8

**DOI:** 10.7150/jca.40592

**Published:** 2020-03-04

**Authors:** Huishan Wang, Li Wang, Lingyu Tang, Jing Luo, Hao ji, Wen Zhang, Jian Zhou, Quanpeng Li, Lin Miao

**Affiliations:** 1 Nanjing Medical University, 101 Longmian Avenue, Jiangning District, Nanjing 211166, Jiangsu Province, China.; 2 Taizhou hospital of traditional Chinese medicine, 86 Jichuandong Road, Hailing District, Taizhou 225300, Jiangsu Province, China.; 3 Department of Thoracic Surgery, Jiangsu Cancer Hospital, Jiangsu Institute of Cancer Research, The Affiliated Cancer Hospital of Nanjing Medical University, Nanjing, China; 4 Medical Center for Digestive Diseases, The Second Affiliated Hospital of Nanjing Medical University, 121 Jiangjiayuan, Nanjing 210011, Jiangsu Province, China.

**Keywords:** cholangiocarcinoma, SNHG6, miR-101-3p, E2F8, ceRNA

## Abstract

Cholangiocarcinoma (CCA) development is an extremely complex process with alterations occurring in numerous genes. SNHG6, a validated lncRNA, has been reported to regulate the expression of multiple tumor-related genes in hepatocellular carcinoma, colorectal cancer and breast cancer. Here, we elucidated the function and possible molecular mechanisms of SNHG6 in human CCA cells. Our results proved that the expression SNHG6 was upregulated in CCA tissues and cell lines. Ectopic expression of SNHG6 promoted cell proliferation, cell cycle progression, migration, and angiogenesis in CCA cells, whereas knockdown of SNHG6 repressed these cellular processes. Further mechanistic studies revealed that SNHG6 could compete with the transcription factor E2F8 to bind with miR-101-3p, thus affecting E2F8 expression. Taken together, these results provided a comprehensive analysis of the role of SNHG6 in CCA cells and offered important clues to understand the key roles of competing endogenous RNA (ceRNA) mechanisms in human cholangiocarcinoma.

## Introduction

Cholangiocarcinoma (CCA) is the second most common primary liver tumor, and accounts for 10%-15% of all hepatobiliary malignancies^1^. It is a malignancy arising from the intra or extrahepatic biliary epithelium, which is characterized with late diagnosis and fatal outcome. Surgery is still the only potentially curative treatment option for CCA but the prognosis of CCA patients is poor, with a five-year overall rate less than 35%^2^. The molecular etiology of CCA is complicated and multiple genomic alterations function in its progression. Therefore, it is becoming urgent to search for reliable molecules involving in the progression and development of CCA.

Long noncoding RNA (lncRNAs) are non-protein coding transcripts longer than 200 nucleotides and have been reported to participate in a variety of biological processes,including regulation of gene expression, subcellular architecture, and stabilization of protein complexes^3^. Mechanistically, lncRNAs sponge many different types of miRNAs, acting as competing endogenous RNAs (ceRNAs) to display its function. Human SNHG6 (Ensembl: ENSG00000245910) is the housekeeping gene of the 5′TOP family, which can encode two kinds of noncoding RNAs; namely, U87 C/D box snoRNA, synthesized by the second intron; and SNHG6 RNA, encoded by exons. SNHG6 is located in chromosome 8q13.1 and its transcription products consist of five transcripts (i.e., SNHG6-201 to SNHG6-205). According to previous studies, SNHG6 is overexpressed in some cancer tissues and cancer cell lines, including hepatocellular carcinoma (HCC)^4^, colorectal cancer (CRC)^5^, ^6^, lung adenocarcinoma (LUAD)^7^, and breast cancer (BC)^8^. What's more, upregulated SNHG6 is related to advanced tumor progression and short survival in these tumor patients. Functionally, SNHG6 participates in cell proliferation, migration, invasion and apoptosis *in vitro*, and promoted tumor growth *in vivo*. However, the biological role and underlying molecular mechanism of SNHG6 in CCA remain largely unknown.

In this study, SNHG6 was identified to be upregulated in CCA and associated with overall survival of CCA patients. We also revealed that downregulation of SNHG6 could inhibit proliferation, metastasis and angiogenesis in CCA cells. In terms of mechanism, SNHG6 served as a ceRNA by binding to miR-101-3p, thereby regulating the expression of E2F8. Overall, our results demonstrated that SNHG6 functioned as an oncogene in CCA cells by increasing the expression of E2F8 through sponging miR-101-3p.

## Methods and materials

### Clinical samples and Cell lines

CCA (n=22) and normal tissues (n=14) were obtained by surgical operations from Second Affiliated Hospital of Nanjing Medical University from June 2014 to July 2018. This study was approved by Second Affiliated Hospital of Nanjing Medical University Review Board. All patients provided signed informed consent. All tissues were resected and put in liquid nitrogen rapidly. All patients were followed up by interview, telephone call and network communication. Overall survival (OS) was defined as the interval between surgery and death or the interval between surgery and the last observation for surviving patients. All the clinical experiments were approved by the research ethics committee of Nanjing Medical University (Nanjing, Jiangsu, PRC).

The CCA cell lines (HCCC-9810 and RBE) and HIBEpiC were obtained from the Institute of Biochemistry and Cell Biology of the Chinese Academy of Sciences (ICBC, Shanghai, China). HCCC-9810 and RBE cell lines were cultured in RMPI-1640, while HIBEpiC in DMEM (Gibco, ThermoFisher, USA), all containing 10% fetal bovine serum (FBS, ScienCell, Carlsbad, CA, USA), 1% streptomycin (Hyclone), and 1% penicillin (Hyclone) and cells were cultured in cell incubator (37℃, 5% CO2). When the cell confluence reached 80-90%, the cells were digested by trypsinase (Gibco, ThermoFisher, USA).

### RNA extraction and qRT-PCR

TRIzol reagent (Invitrogen, Carlsbad, USA) was used to isolate the total RNA from the cultured cells and tissue samples, and miRNA was extracted by miRcute miRNA Isolation Kit (TIANGEN, Beijing, China). The reverse transcription reaction of total RNA was conducted using PrimeScript^TM^ RT reagent Kit with gDNA Eraser (Takara, RR047A, Japan), whereas miRcute Plus miRNA First-Strand cDNA Kit (TIANGEN, Beijing, China) was used for the reverse transcription reaction of miRNA. The obtained cDNA from total RNA was subjected to PCR amplification using SYBR green (Takara, RR420A, Japan) with ABI 7500 Real-Time PCR System (ThermoFisher), while the cDNA from miRNA was amplified with miRcute Plus miRNA qPCR Kit (SYBR Green, TIANGEN, Beijing, China). U6 and β-actin were used as endogenous controls for miRNAs and mRNAs, respectively. 2-^ΔΔCt^ method was used to calculate the relative expression. The primers used were listed in Table S1.

### Transfection

When the cells in 6-well plates is approximately 40% of confluence, small interfering RNAs (or miRNA inhibitor or miRNA mimics) were transfected into cells using lipofectamine 3000 (ThermoFisher, USA) according to the product instruction. miR-101-3p mimics, miR-101-3p inhibitor and negative control miRNA (Control) were purchased from GenePharma (Shanghai, China). All the mentioned sequences were listed in Table S2.

### Cell proliferation assays

Cell viability was monitored with CCK8 kit (CCK8, Dojindo, Japan) following the producer's suggestions. The HCCC-9810 and RBE cells transfected with si-SNHG6 or si-NC (scramble negative control) (2000 cells/well) were cultivated in five 96-well plates with six replicate wells. 10 ul CCK-8 solution was added to each well, incubated for 2 h, and then assayed using a microplate reader with a wavelength of 450 nm. For the colony formation assay, cells were seeded into six-well plates with 500 cells/well and cultured for 10-14 days. Then, the cells were stained with a 10% crystal violet solution (Sigma, NY, USA). Visible colonies were counted. All experiments were repeated at least three times.

### Cell migration and wound healing assays

Cell migration ability was determined using transwell chambers (24-well insert, 8 μm, Corning, NY, USA). Briefly, 4×10^4^ transfected cells were seeded in the upper chamber with 300 μl serum-free medium. And 600 μl DMEM containing 20% FBS was added to the lower chamber. After incubation for 24 h, the cells that had passed through the bottom membrane were stained with a 10% crystal violet solution (Sigma, NY, USA). The migrated cells were counted under the microscope. For wound healing assay, an artificial wound was created in cells with a 10 μl plastic pipette tip after transfection. The wound closure was imaged under a microscope at 0 and 36 h. All experiments were repeated at least three times.

### Tube formation assay

HUVECs cells (~4×10^5^) were suspended by the mixture of tumor-conditioned medium (TCM, 300μl) and ECM containing 10% FBS (300μl), and then plated into a 24-well plate precoated with matrigel (200 μl per well, BD Biosciences, USA). Tube formation was observed after 8 h incubation at 37℃ and was imaged with a computer-assisted inverted microscope (Nikon, Japan), the number of tube branches was counted by image J software. The experiment was repeated at least three times.

### Flow cytometric analysis of cell cycle distribution

After transfected with siRNAs for 48 h, the cells were harvested, and washed with ice-cold PBS, then fixed with 70% ethanol overnight at -20°C. The ethanol-suspended cells were centrifuged and stained with PI staining solution for 10 min in the dark at 37°C.A FACS Calibur flow cytometer was used to detect cell cycle distribution. The percentage of cells in G1, S, and G2-M phases were counted and compared. The experiment was repeated at least three times.

### Zebrafish xenografts

HCCC-9810 cells transfected with si-SNHG6 or negative control were labelled with CellTracker™ CM-DiI (Molecular probes, Invitrogen, Waltham, MA) according to the instruction. Two hundred cells/embryo were then injected in the yolk sac of Tg (fli1: EGFP) zebrafish larvae 48 h after fertilization. After incubation at 35℃ for 4 days, larvae were analyzed for human CCA cells dissemination by fluorescence microscopy. All animal experiments were conducted in accordance with the guidelines and rules for the protection and utilization of experimental animals of Nanjing Medical University.

### RNA sequencing

Total RNA from the HCCC-9810 cells with SNHG6 knockdown or control were separated and quantified. The concentration of each specimen was measured with NanoDrop 2000 (Thermo Scientific, USA). Further RNA sequencing was performed by the Beijing Biomarker Institute using the Illumina Genome Analyzer (Beijing, China). The clustering of the index-coded samples was performed on a cBot Cluster Generation System using TruSeq PE Cluster Kit v4-cBot-HS (Illumia) according to the manufacturer's instructions. After cluster generation, the library preparations were sequenced on an Illumina Hiseq Xten platform and paired-end reads were generated.

### KEGG pathway enrichment analysis

KEGG is a database resource for understanding high-level functions and utilities of the biological system. We used KOBAS software to test the statistical enrichment of differential expression genes in KEGG pathways. Cluster Profiler R packages were used to find KEGG pathway that are significantly enriched compared to the entire genome background. Pathways with Q value ≤ 0.05 are defined as Pathways that are significantly enriched in differentially expressed genes.

### Subcellular fractionation address

The division of nuclear as well as cytosolic fractions was constructed with the PARIS Kit (Life Technologies, Carlsbad, CA, USA) following the producer's guides. Briefly, cells were washed twice using pre-chilled PBS, and then lysed. After centrifugation at 300 ×g for 5 min, the supernatant (cytoplasmic fraction) was collected, and the remaining pellet, which was considered to be the nuclear fraction, was collected after an additional five washes using PBS. The experiment was repeated at least three times.

### Western blot analysis

Total cellular protein was extracted using RIPA lysis buffer containing protease inhibitor cocktail (Beyotin, China). The extracted proteins were separated by SDS-PAGE and transferred onto PVDF membranes (Millipore, NY, USA), which were subsequently blocked with a 5% solution of skim milk for 1 h. The membranes were then incubated with primary antibodies at 4 °C overnight, followed by secondary antibody incubation for 1 h at room temperature. The proteins were visualized with a SuperLumia ECL HRP Substrate Kit (Millipore, NY, USA) and detected using an LAS4000 chemiluminescence detection system (Fuji, Tokyo, Japan). The primary antibodies used were as follows: anti-β-actin (1:1000, Beyotin, AA128) and anti-E2F8 (1: 500, sc-514064, Santa-Cruz Technology). An anti-β-actin antibody was used as an internal control.

### Luciferase assay

Luciferase activity was measured using a Dual Luciferase Assay Kit (Promega, Madison, WI, USA) according to the manufacturer's instructions. Briefly, SNHG6 luciferase reporter plasmid and Renilla luciferase plasmid as internal reference were transiently transfected into HCCC-9810 cells using X‐treme GENE HP DNA transfection reagent (Roche). Luciferase activity was detected using the Dual‐Luciferase Reporter Assay System (Promega, Madison, WI) 48 hours after transfection.

### Statistical analysis

Stata 13.0 and R packages were used for data analysis. GraphPad Prism 7.0 and Image J were used for image editing. Paired t test was performed to compare quantitative data. Classification data were compared using chi-square test. Survival analysis were evaluated using the Kaplan-Meier method, and differences in survival distributions were assessed by the Log-rank test. P <0.05 was considered statistically significant. *p <0.05, **p <0.01.

## Results

### SNHG6 was upregulated in CCA tissues and associated with poor overall survival in CCA patients

**Figure 1:**
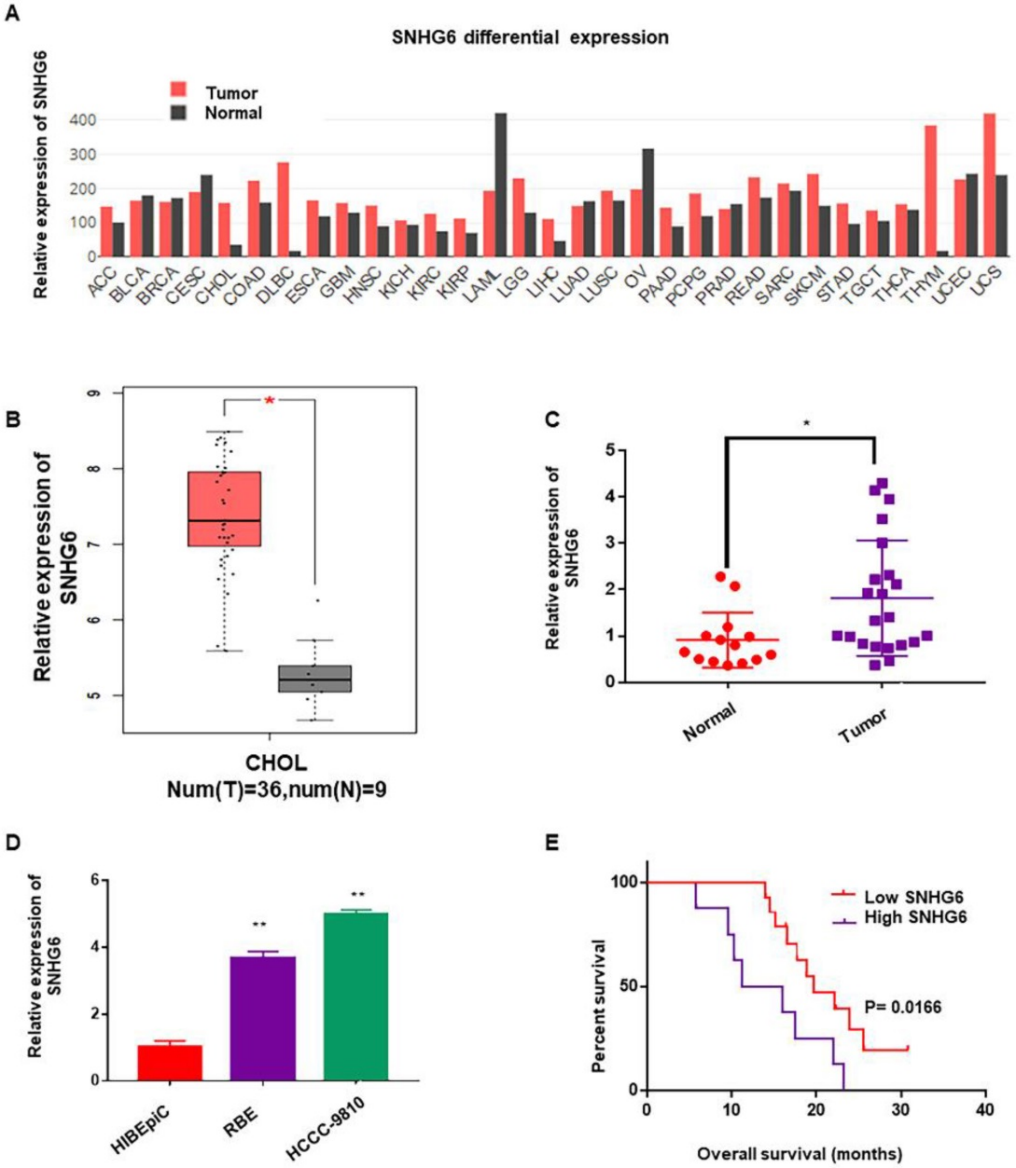
** SNHG6 was overexpressed in CCA tissues and cells, and associated with poor overall survival in CCA patients.** (A) SNHG6 expression (TPM) in different human malignancies and their matched normal tissues from the GEPIA software (http://firebrowse.org). (B) SNHG6 expression (Log_2_(TPM+1) in CCA tissues (n=36) compared with noncancerous tissues (n=9) from TCGA database analyzed with GEPIA software. (C) The relative expression (normalized to β-actin) of SNHG6 in CCA tissues (n=22) and adjacent normal tissues (n=14). (D) Relative SNHG6 expression in two CCA cell lines (HCCC-9810 and RBE) and one normal biliary epithelial cell line HIBEpiC. (E) Kaplan-Meier survival plots showed that high SNHG6 expression was correlated with poor OS in CCA patients (n=22, p=0.0166).

To determine whether SNHG6 was abnormally expressed in different kinds of cancer, we first analyzed the expression of SNHG6 by RNA-Seq data (TCGA) from the bioinformatics software GEPIA (http://gepia.cancer-pku.cn/)^9^. As shown in Figure 1A, SNHG6 expression was upregulated in the majority of malignant tumors than in normal tissues (Fig. 1A). Subsequently, we analyzed SNHG6 levels in CCA tissues and normal tissues in this software, and results showed that SNHG6 was overexpressed in CCA tissues (n=36) than in normal tissues (n=9) (Fig. 1B). To further confirm this finding, we measured SNHG6 expression in 22 CCA tissues and 14 normal tissues that were resected from patients at our hospital. qRT-PCR analysis also showed that SNHG6 was highly expressed in CCA tissues (Fig. 1C). In addition, the expression abundances of SNHG6 in two CCA cell lines (HCCC-9810 and RBE) was higher than normal bile duct epithelial cell line (HIBEpiC) (Fig. 1D). What's more, high SNHG6 expression levels were correlated with poor overall survival in CCA patients (Fig. 1E).

### SNHG6 promoted CCA cells proliferation, migration and HUVECs tube formation

**Figure 2:**
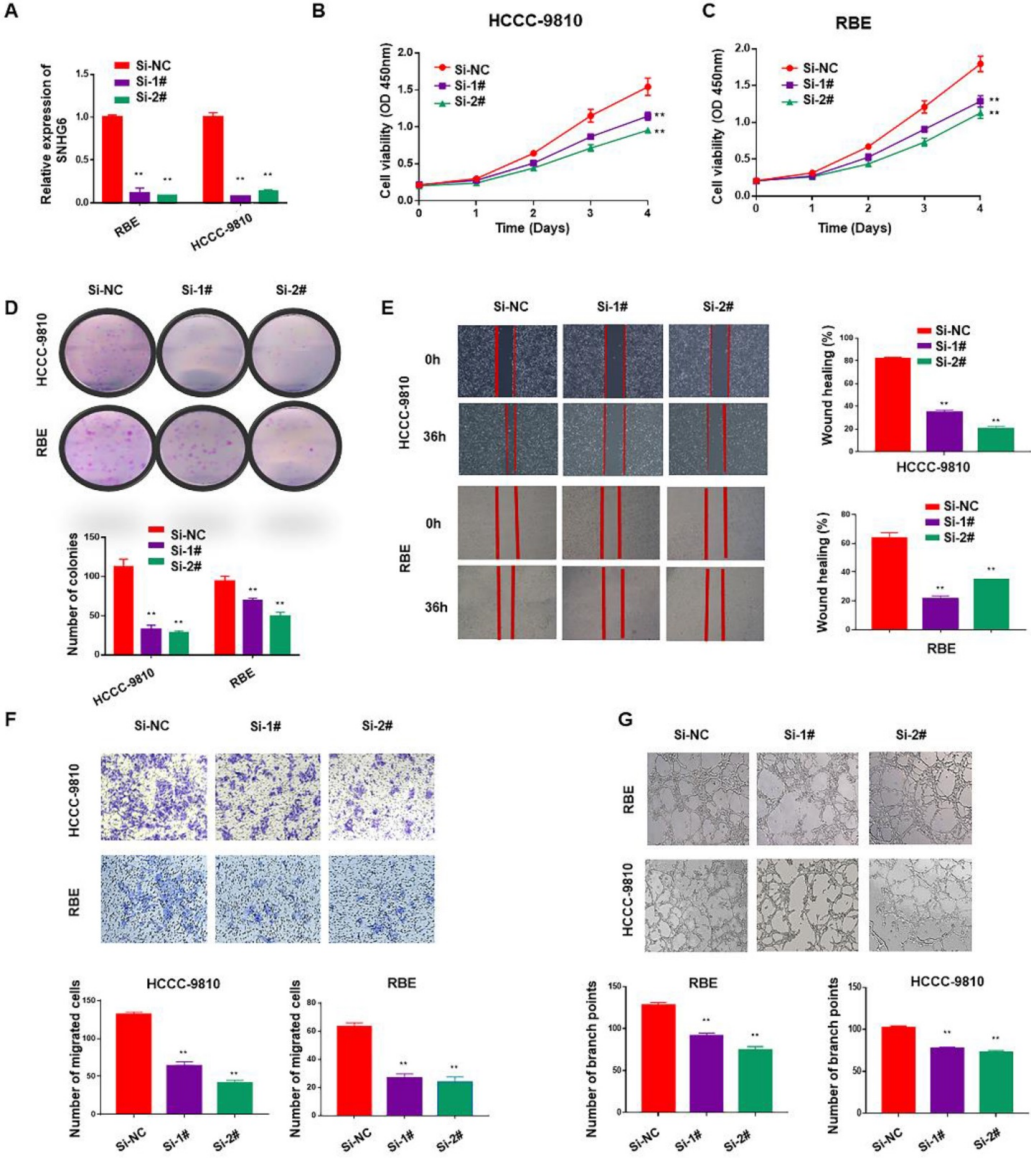
** SNHG6 promoted cell proliferation, migration and HUVECs tube formation in CCA cells *in vitro*.** (A) qRT-PCR analysis of SNHG6 expression following SNHG6 knockdown with siRNA in HCCC-9810 and RBE cell lines. (B-C) CCK8 assays were performed to evaluate the relative cell viability at different times in the HCCC-9810 and RBE cells after transfection with si-SNHG6 or si-NC as control. (D) In colony formation assay, colony numbers of si-SNHG6 group were less than si-NC group. (E) Wound healing assay was used to evaluate the effect of si-SNHG6 on motility in the CCA cell lines. (F) Transwell assay indicated that knockdown SNHG6 inhibited the migration of CCA cells compared with control group. (G) HUVECs were cultured in tumor-conditional- medium (TCM) derived from HCCC-9810 or RBE cells transfected with si-SNHG6 or si-NC, the relative number of tube branches were measured in random 5 photographic fields. Data were shown as mean ± SD. * p < 0.05, ** p < 0.01.

To identify the function of SNHG6 in CCA, we firstly used two specific siRNAs to silence endogenous SNHG6 in HCCC-9810 and RBE cells. After 48 hours of transfection, SNHG6 expression was dramatically downregulated in HCCC-9810 and RBE cells than in the control cells (Fig. 2A). Cell proliferation ability was determined by CCK-8 and colony formation assays. Silencing SNHG6 prominently reduced cell growth rates (Fig. 2B and C) and the number of foci formed (Fig. 2D) relative to the control groups. Next, we investigated the influence of SNHG6 on CCA metastasis by wound healing and transwell assays. The results indicated that wound closure was slower in SNHG6 knockdown CCA cells than that in the control group (Fig. 2E), and silencing SNHG6 in CCA cells (HCCC-9810 cells and RBE cells) resulted in poorer cell migration capacity (Fig. 2F). What's more, we observed that TCM derived from si-SNHG6 CCA cells could suppress HUVECs tube formation compared with that from si-NC group (Fig. 2G).

### SNHG6 might exerted its biological role in CCA cells via modulating E2F8

**Figure 3:**
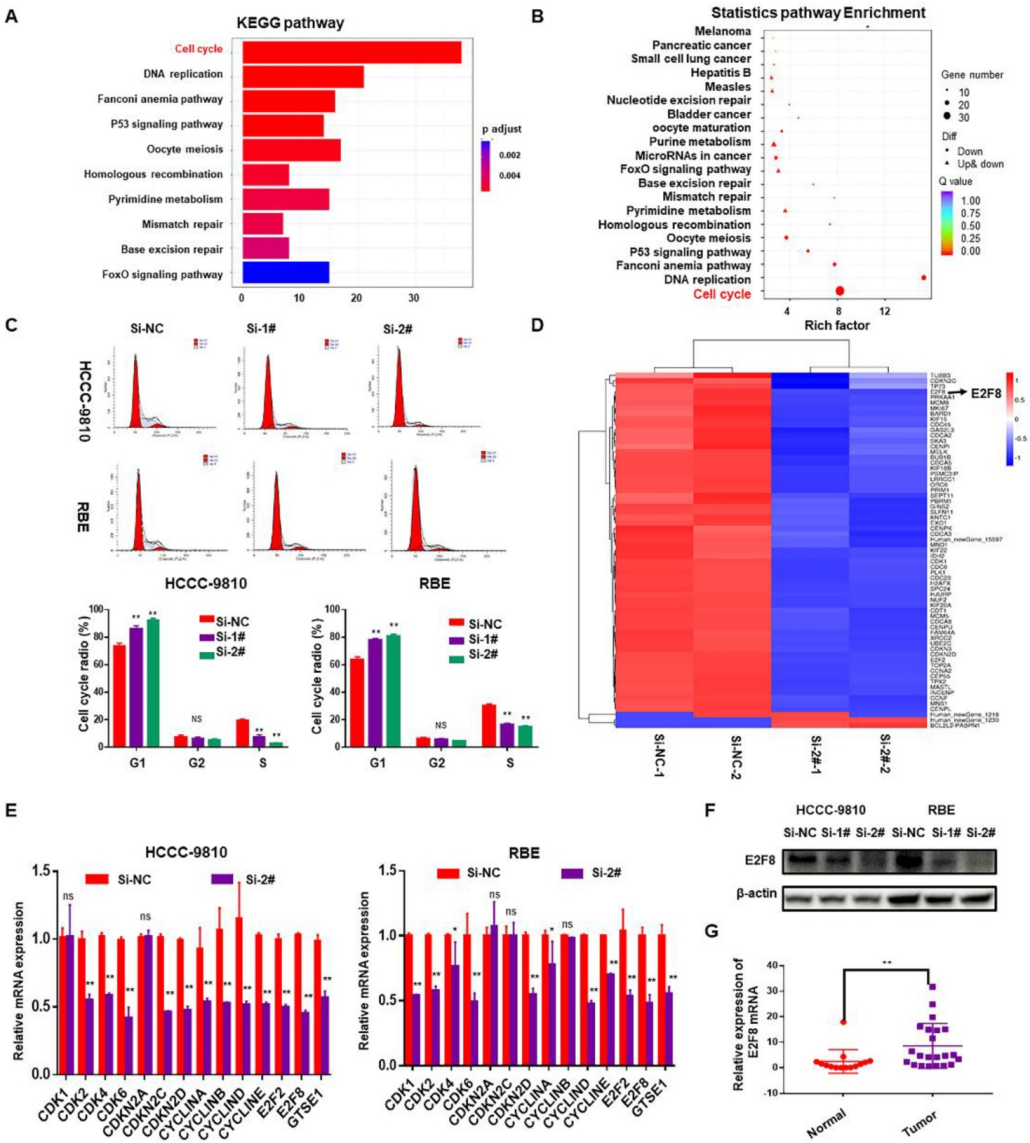
** SNHG6 might exerted its biological role in CCA cells via modulating E2F8.** mRNA expression profile was analyzed after SNHG6 knockdown in HCCC-9810 cell. (A). KEGG pathway enrichment analysis of the differentially expressed genes presented by Q value. (B). Rich Factor was also used to analyze the differentially expressed genes. The larger the Rich Factor, the greater the degree of enrichment. (C). Flow cytometry assay was used to analyze the cell cycle, and cell cycle was arrested in G0/G1 phase after SNHG6 was knocked down. (D). Heat map of the differential expressed mRNAs involved in cell cycle pathway. (E). qRT-PCR and (F). western blot was used to measure the expression of E2F8 in HCCC-9810 and RBE cell line after transfected with si-SNHG6 or si-NC. (F). qRT-PCR was used to detect the relative expression of E2F8 in CCA tissues (n=22) and adjacent normal tissues (n=14). Data were shown as mean ± SD. * p < 0.05, ** p < 0.01.

The above data suggested that SNHG6 is overexpressed in CCA tissues and cell lines and is related to the progression of CCA cells. Accordingly, we next performed gene expression profiling of control and SNHG6 knockdown HCCC-9810 cells. Knockdown of SNHG6 resulted in the differential expression of 781 genes (p<0.05, log_2_(fold change)>2) (Table S3). Pathway enrichment analysis of these differential genes using the KEGG public database with two evaluation methods: Q value and Rich Factor^10^. The analysis suggested that the biological processes with high correlation of mRNA expression profile changes caused by SNHG6 knockdown may be highly related to cell cycle regulation of CCA cells (Fig.3A and 3B). Therefore, flow cytometry was performed to validate this finding, and the results showed that SNHG6 knockdown significantly arrested cycle progression by increasing the G0/G1 phase cell population and decreasing the S phase cell population (Fig. 3C). Furthermore, a heat map was drawn to show the differential expression of genes involved in cell cycle pathway in the SNHG6-knockdown HCCC-9810 cells (Fig. 3D, p<0.05, log_2_FC>1.5 or <-1.5). Quantity RT-PCR was performed to confirm the expression of some cell-cycle genes both in HCCC-9810 and RBE cells, and the results were mainly consistent with the above sequencing results (Fig.3E). Previous studies have shown that E2F transcription factor 8 (E2F8) plays an essential role in regulation of cellular functions, including cell cycle, cell proliferation, cell survival, DNA damage and angiogenesis^11^. Western blot showed that SNHG6 knockdown could also reduce the protein expression of E2F8 (Fig. 3F). And qRT-PCR showed that E2F8 was upregulated in CCA tissues (Fig. 3G). Taken together, E2F8 may be a potential downstream of SNHG6.

### SNHG6 increased the expression of E2F8 through binding to miR-101-3p in CCA cells

**Figure 4:**
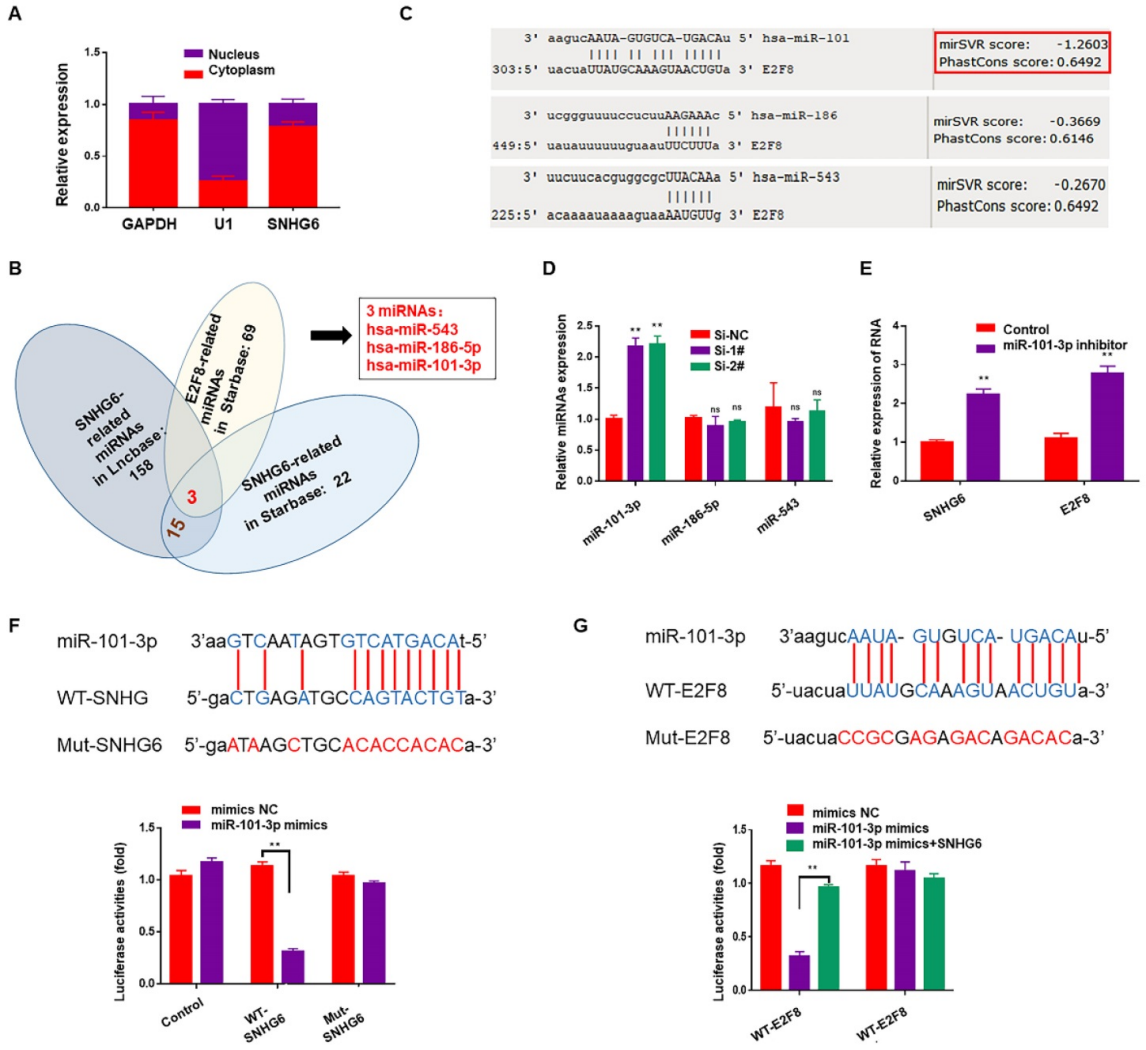
** SNHG6 increased the expression of E2F8 through binding to miR-101-3p in CCA cells.** (A). Subcellular fractionation assays were used to examine SNHG6 localization in HCCC-9810 cells, and SNHG6 was localized mainly in the cytoplasm. (B). Venn diagram for miRNA screening. (C). miR-101-3p had the most potential to sponge E2F8 3′-UTR by MirSVR. (D). qRT-PCR assays were performed to assess the effect of SNHG6 on these three miRNAs expression in transfected HCCC-9810 cells, and the expression of miR-101-3p was upregulated by silencing SNHG6. (E). miR-101-3p inhibitor could both upregulate the expression of SNHG6 and E2F8 in RNA level. (F). Luciferase activity was detected using the dual luciferase assay. The results showed a decrease in luciferase activity in cells transfected with WT-SNHG6 and miR-101-3p mimics. (G). Luciferase activity in cells transfected with WT-E2F8 and miR-101-3p mimics was decreased, which could be partly reversed by overexpressing SNHG6.

Subcellular fractionation assays showed that SNHG6 was mainly located in cytoplasm (Fig. 4A). Cytoplasmic lncRNAs can bind directly to miRNA and function as sponges or ceRNAs to control the availability of miRNA for binding to their target mRNAs. To examine whether SNHG6 could function as a miRNA sponge to regulate E2F8, Starbase (http://starbase.sysu.edu.cn) ^12^and DIANA-LncBasev2 **(**http://www.microrna.gr/LncBase) ^13^were used to predict the potential target miRNAs of SNHG6, and 15 miRNAs were found after intersection. Along with the miRNAs predicted to be the upstream of E2F8 in Starbase, we finally got three miRNAs: has-miR543, has-miR-186-5p and has-miR-101-3p (Fig.4B). Then another database (http://www.microRNA.org) ^14^was used to judge the binding possibilities of each miRNA to E2F8 3′-UTR and miR-101-3p had the most potential to sponge E2F8 3′-UTR (Fig. 4C). Furthermore, miR-101-3p expression was upregulated by silencing SNHG6 (Fig. 4D), and also miR-101-3p inhibitor could upregulate the expression of SNHG6 and E2F8 in RNA level (Fig. 4E). Based on the above findings, we hypothesized that SNHG6 functioned as a competing endogenous RNA to regulate E2F8 expression by sponging miR-101-3p in CCA cells. Then, we utilized a dual-luciferase reporter system to confirm the relationship between SNHG6 and miR-101-3p. The results revealed that the ectopic expression of miR-101-3p mimics decreased reporter luciferase activity of WT-SNHG6 and WT-E2F8-3'-UTR in HCCC-9810 cells, whereas no significant luciferase activity was observed in MUT-SNHG6 and MUT-E2F8-3'-UTR cells. Besides, in the cells co-transfected with WT-E2F8-3'-UTR and miR-101-3p mimics, overexpressing SNHG6 could partly rescued the decreasing luciferase activity (Fig. 4F-G).

### The SNHG6/miR-101-3p/E2F8 axis played a critical role in the cell proliferation and angiogenesis of CCA

**Figure 5:**
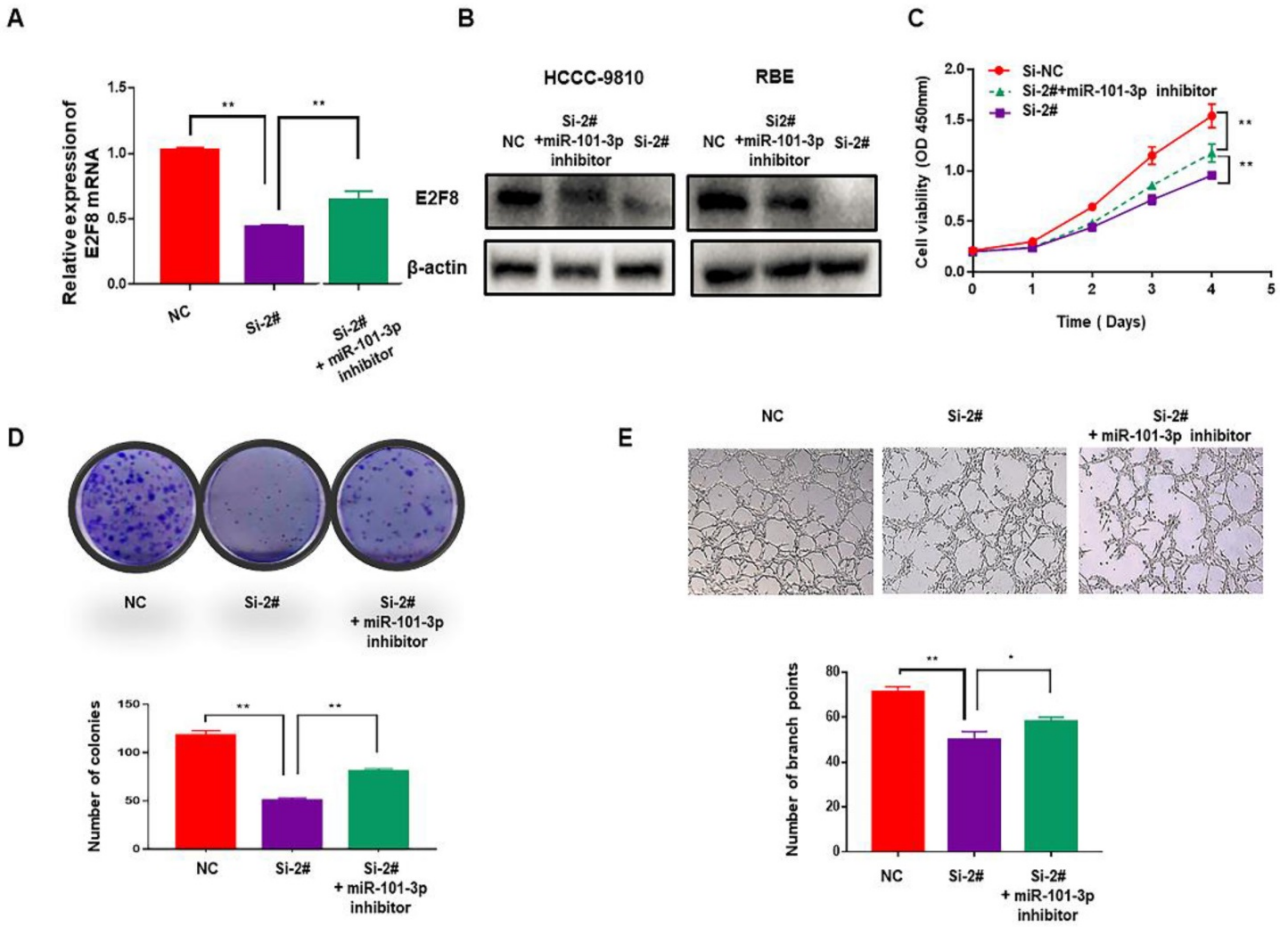
** The SNHG6/miR-101-3p/E2F8 axis is critical for CCA cell proliferation and angiogenesis.** (A). qRT-PCR revealed that the decreasing expression of E2F8 caused by silencing SNHG6 could be partly rescued by miR-101-3p inhibitor. (B). The expression of E2F8 in protein level was also rescued by miR-101-3p inhibitor in HCCC-9810 cells. (D). CCK8 and (E). colony formation assays indicated that miR-101-3p partially alleviated the rapid proliferation ability of CCA cells caused by SNHG6. (F). HUVECs were cultured in tumor-conditional- medium (TCM) derived from HCCC-9810 cells transfected with si-SNHG6, si-SNHG6+miR-101-3p inhibitor or NC, and angiogenesis inhibition induced by Si-SNHG6 could be partly reversed by miR-101-3p inhibitor.

To verify whether SNHG6 induces cell proliferation and angiogenesis in CCA cells through targeting the miR-101-3p/E2F8 axis, miR-101-3p inhibitor was used. After downregulating miR-101-3p, decreased expression of E2F8 induced by si-SNHG6 was rescued both in mRNA and protein level (Fig. 5A and 5B). Moreover, CCK8 and colony formation assays indicated that proliferation inhibition induced by knockdown of SNHG6 could be partly reversed by miR-101-3p inhibitor (Fig. 5C and 5D), and so as angiogenesis (Fig. 5E). These findings were consistent with our hypothesis and indicated that SNHG6 promoted cell cycle, proliferation and angiogenesis in CCA cells by sponging miR-101-3p to prevent E2F8 inhibition.

### SNHG6 promoted CCA tumor growth and metastasis in zebrafish xenograft model

**Figure 6:**
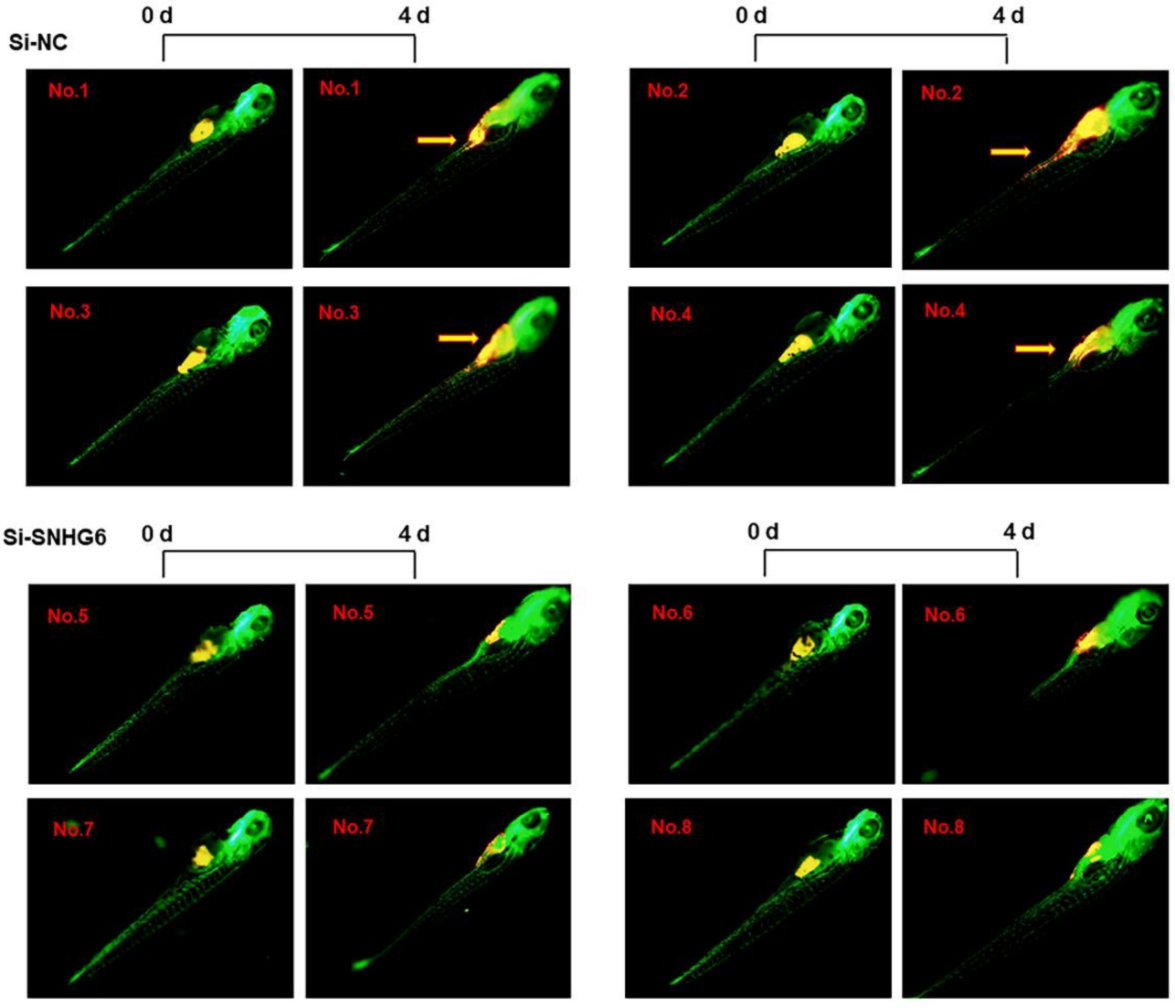
** SNHG6 promotes cell proliferation, migration in CCA cells *in vivo*.** Representative images of zebrafish invaded or not by yellow-fluorescent human CCA cells are shown. Compared with Si-NC group, silencing SNHG6 group had smaller cell mass and lower migrate rate. Yellow arrows point to migrated CCA cell.

To investigate the ability of SNHG6 to enhance human choangiocarcinoma proliferation and invasion *in vivo*, HCCC-9810 cells transfected with si-SNHG6-2# or si-NC were implanted in the yolk sac of zebrafish larvae, and CCA cell invasion was evaluated 4 days after injection. As shown in Figure 6, SNHG6 knockdown resulted in smaller size of cell mass and a significant lower dissemination from the primary site of injection and colonization into distal parts of the larvae compared with the control clone Mneo.

## Discussion

Recently, lncRNAs have gained great attention as its role in CCA^15^. Several known lncRNAs were reported to be dysregulated in CCA tissues and involved in the CCA development and progression, such as DANCR^16^, PVT1^17^, and RMRP^18^. Recent studies have reported that SNHG6 is highly expressed in variety of cancer tissues (except CCA) and overexpressed SNHG6 reduces cell apoptosis but promotes cell cycle, cell migration, invasion, EMT, and chemoresistance. SNHG6 exerts its function mainly by serving as miRNA sponge to antagonize the connections between multiple tumor suppressor miRNAs and their target mRNAs^19^-^23^. In this study, we analyzed public database and found that SNHG6 was also upregulated in CCA cell lines and tissues, and functional investigations showed that SNHG6 silencing inhibited cell proliferation, migration, and HUVECs tube formation, which indicated that SNHG6 functioned as an oncogene in CCA.

By high-throughput sequencing further verification, we found that SNHG6 played its oncogenic role in CCA by regulating cell cycle process, especially via regulating E2F8. Several studies have proved that E2F8 is upregulated in HCC^24^-^26^, and causes enhanced cell proliferation, promoted colony formation and tumorigenesis. Mechanistic analyses indicated that E2F8 could bind to regulatory elements of cyclin D1, thus regulating its transcription and promoting accumulation of S-phase cells in HCC^27^. E2F8 is also reported to be overexpressed in clinical patients with lung cancer^28^, breast cancer 29, prostate cancer^30^, and papillary thyroid cancer^31^. Besides, high expression of E2F8 is associated with worse recurrence free survival (RFS) in ER-positive breast cancer by conferring cisplatin resistance 32. The specific mutant of E2F8 in the extra-embryonic trophoblastic cells also can lead to a poor structure of the placental vascular network, indicating that E2F8 is necessary for angiogenesis^33^. The oxygen and nutrients supplied by the vasculature are crucial for cell function and survival, especially for aberrant proliferative cancer cells. In order to progress malignant lesions to a larger size, incipient neoplasms must develop angiogenic ability. Meanwhile, angiogenesis and proliferation could help cancer cells acquire the ability of invasion and metastasis^34^. In the case, we validated that SNHG6 promotes cell growth and angiogenesis in CCA mainly via E2F8, which is also highly expressed in CCA tissues. Knockdown of SNHG6 induced G1/S cell cycle arrest and inhibited angiogenesis. Additionally, the expression of E2F8 both in mRNA and protein levels decreased when SNHG6 was silenced.

Increasing studies have demonstrated that lncRNAs could regulate mRNA levels by competing with miRNAs and acting as ceRNAs. It has been reported that SNHG6 could function as a sponge for miR-101-3p in CCA^35^, ^36^, and miR-101-3p inhibits CCA angiogenesis through targeting vascular endothelial growth factor (VEGF)^36^. In our study, we found that miR-101-3p inhibitor could upregulate the expression of SNHG6 and E2F8 in CCA cells, and luciferase assay indicated that SNHG6 could bind to miR-101-3p and miR-101-3p could also bind to E2F8 3′-UTR. Moreover, inhibition of miR-101-3p could abolish the biological effect mediated by SNHG6 knockdown *in vitro*. These data supported the role of SNHG6 as a sponge of miR-101-3p in CCA, and E2F8, released by miR-101-3p, plays a vital role in proliferation and angiogenesis of CCA.

Additionally, using zebrafish xenotransplantation models, we demonstrate that upregulated SNHG6 is associated with the increased ability of CCA cells proliferation and invasion *in vivo*. This experimental model emerged as a powerful tool in the cancer research field to monitor, by means of *in vivo* live imaging, cancer-associated features such as cell invasion, proliferation^37^ and the interaction with immune cells in tumor microenvironment^38^.

In summary, we identified a ceRNA model involving the SNHG6/miR-101-3p/E2F8 axis that is pivotal for cell cycle progression and angiogenesis in CCA cells. Our findings uncover the role of SNHG6 as a regulator of CCA progression, and sheds new light on understanding of lncRNA-mediated malignancy progression. It also suggested that SNHG6 might serve as a potential prognostic biomarker and a promising therapeutic target for CCA.

## Supplementary Material

Supplementary tables 1-2.

Supplementary table 3.

## References

[1] Bergquist A, von Seth E (2015). Epidemiology of cholangiocarcinoma. Best Practice & Research Clinical Gastroenterology.

[2] Maithel SK, Gamblin TC, Kamel I (2013). Multidisciplinary approaches to intrahepatic cholangiocarcinoma. CANCER-AM CANCER SOC.

[3] Gutschner T, Richtig G, Haemmerle M (2018). From biomarkers to therapeutic targets-the promises and perils of long non-coding RNAs in cancer. Cancer Metastasis Rev.

[4] Chang L, Yuan Y, Li C (2016). Upregulation of SNHG6 regulates ZEB1 expression by competitively binding miR-101-3p and interacting with UPF1 in hepatocellular carcinoma. CANCER LETT.

[5] Wang X, Lai Q, He J (2019). LncRNA SNHG6 promotes proliferation, invasion and migration in colorectal cancer cells by activating TGF-beta/Smad signaling pathway via targeting UPF1 and inducing EMT via regulation of ZEB1. INT J MED SCI.

[6] Zhu Y, Xing Y, Chi F (2018). Long noncoding RNA SNHG6 promotes the progression of colorectal cancer through sponging miR-760 and activation of FOXC1. Onco Targets Ther.

[7] Liang R, Xiao G, Wang M (2018). SNHG6 functions as a competing endogenous RNA to regulate E2F7 expression by sponging miR-26a-5p in lung adenocarcinoma. BIOMED PHARMACOTHER.

[8] Lv P, Qiu X, Gu Y (2019). Long non-coding RNA SNHG6 enhances cell proliferation, migration and invasion by regulating miR-26a-5p/MAPK6 in breast cancer. BIOMED PHARMACOTHER.

[9] Tang Z, Li C, Kang B (2017). GEPIA: a web server for cancer and normal gene expression profiling and interactive analyses. NUCLEIC ACIDS RES.

[10] Kanehisa M, Furumichi M, Tanabe M (2017). KEGG: new perspectives on genomes, pathways, diseases and drugs. NUCLEIC ACIDS RES.

[11] Lv Y, Xiao J, Liu J (2017). E2F8 is a Potential Therapeutic Target for Hepatocellular Carcinoma. J CANCER.

[12] Li JH, Liu S, Zhou H (2014). starBase v2.0: decoding miRNA-ceRNA, miRNA-ncRNA and protein-RNA interaction networks from large-scale CLIP-Seq data. NUCLEIC ACIDS RES.

[13] Paraskevopoulou MD, Vlachos IS, Karagkouni D (2016). DIANA-LncBase v2: indexing microRNA targets on non-coding transcripts. NUCLEIC ACIDS RES.

[14] Betel D, Wilson M, Gabow A (2008). The microRNA.org resource: targets and expression. NUCLEIC ACIDS RES.

[15] Li J, Huang L, Li Z (2019). Functions and roles of long noncoding RNA in cholangiocarcinoma. J CELL PHYSIOL.

[16] Wang N, Zhang C, Wang W (2019). Long noncoding RNA DANCR regulates proliferation and migration by epigenetically silencing FBP1 in tumorigenesis of cholangiocarcinoma. CELL DEATH DIS.

[17] Yu Y, Zhang M, Liu J (2018). Long Non-coding RNA PVT1 Promotes Cell Proliferation and Migration by Silencing ANGPTL4 Expression in Cholangiocarcinoma. Mol Ther Nucleic Acids.

[18] Tang L, Wang Y, Wang H (2019). Long noncoding-RNA component of mitochondrial RNA processing endoribonuclease is involved in the progression of cholangiocarcinoma by regulating microRNA-217. CANCER SCI.

[19] Lv P, Qiu X, Gu Y (2019). Long non-coding RNA SNHG6 enhances cell proliferation, migration and invasion by regulating miR-26a-5p/MAPK6 in breast cancer. BIOMED PHARMACOTHER.

[20] Ruan J, Zheng L, Hu N (2018). Long noncoding RNA SNHG6 promotes osteosarcoma cell proliferation through regulating p21 and KLF2. ARCH BIOCHEM BIOPHYS.

[21] Wu Y, Deng Y, Guo Q (2019). Long non-coding RNA SNHG6 promotes cell proliferation and migration through sponging miR-4465 in ovarian clear cell carcinoma. J CELL MOL MED.

[22] Zhang M, Duan W, Sun W (2019). LncRNA SNHG6 promotes the migration, invasion, and epithelial-mesenchymal transition of colorectal cancer cells by miR-26a/EZH2 axis. Onco Targets Ther.

[23] Zhu X, Yang G, Xu J (2019). Silencing of SNHG6 induced cell autophagy by targeting miR-26a-5p/ULK1 signaling pathway in human osteosarcoma. CANCER CELL INT.

[24] Baiz D, Dapas B, Farra R (2014). Bortezomib effect on E2F and cyclin family members in human hepatocellular carcinoma cell lines. World J Gastroenterol.

[25] Evangelou K, Havaki S, Kotsinas A (2014). E2F transcription factors and digestive system malignancies: how much do we know?. World J Gastroenterol.

[26] Xanthoulis A, Tiniakos DG (2013). E2F transcription factors and digestive system malignancies: how much do we know?. World J Gastroenterol.

[27] Deng Q, Wang Q, Zong WY (2010). E2F8 contributes to human hepatocellular carcinoma via regulating cell proliferation. CANCER RES.

[28] Park SA, Platt J, Lee JW (2015). E2F8 as a Novel Therapeutic Target for Lung Cancer.

[29] Ye L, Guo L, He Z (2016). Upregulation of E2F8 promotes cell proliferation and tumorigenicity in breast cancer by modulating G1/S phase transition. Oncotarget.

[30] Lee S, Park YR, Kim SH (2016). Geraniol suppresses prostate cancer growth through down-regulation of E2F8. Cancer Med.

[31] Sun J, Shi R, Zhao S (2017). E2F8, a direct target of miR-144, promotes papillary thyroid cancer progression via regulating cell cycle. J Exp Clin Cancer Res.

[32] Tian J, Lin Y, Yu J (2017). E2F8 confers cisplatin resistance to ER+ breast cancer cells via transcriptionally activating MASTL. BIOMED PHARMACOTHER.

[33] Ouseph MM, Li J, Chen HZ (2012). Atypical E2F repressors and activators coordinate placental development. DEV CELL.

[34] Hanahan D, Weinberg RA (2000). The Hallmarks of Cancer. CELL.

[35] Xu Y, Yao Y, Jiang X (2018). SP1-induced upregulation of lncRNA SPRY4-IT1 exerts oncogenic properties by scaffolding EZH2/LSD1/DNMT1 and sponging miR-101-3p in cholangiocarcinoma. J Exp Clin Cancer Res.

[36] Zhang J, Han C, Zhu H (2013). miR-101 inhibits cholangiocarcinoma angiogenesis through targeting vascular endothelial growth factor (VEGF). AM J PATHOL.

[37] White R, Rose K, Zon L (2013). Zebrafish cancer: the state of the art and the path forward. NAT REV CANCER.

[38] Feng Y, Martin P (2015). Imaging innate immune responses at tumour initiation: new insights from fish and flies. NAT REV CANCER.

