# Supplementary material for: Long noncoding RNA SNHG6 promotes proliferation and angiogenesis of cholangiocarcinoma cells through sponging miR-101-3p and activation of E2F8
Source: J Cancer. 2020 Mar 4;11(10):3002–12. doi: 10.7150/jca.40592 (PMC7086249; doi:10.7150/jca.40592)
Supplement: Supplementary file 1 — Supplementary tables 1-2. [file jcav11p3002s1.pdf]

Primers

	Forward primer	Reverse primer
β-actin	TGGCACCCAGCACAATGAA	CTAAGTCATAGTCCGCCTAGAAGCA
SNHG6	TTCTCGGCATCCACATTTGCTC	CAGGCGGGTCTTATGAGAGAGA
CDKN2C	GGGGACCTAGAGCAACTTACT	CAGCGCAGTCCTTCCAAAT
CDKN2D	AGTCCAGTCCATGACGCAG	ATCAGGCACGTTGACATCAGC
CDKN2A	ATGGAGCCTTCGGCTGACT	GTAACTATTCGGTGCGTTGGG
CDK4	ATGGCTACCTCTCGATATGAGC	CATTGGGGACTCTCACACTCT
CDK1	AAACTACAGGTCAAGTGGTAGCC	TCCTGCATAAGCACATCCTGA
CDK2	GTACCTCCCCTGGATGAAGAT	CGAAATCCGCTTGTTAGGGTC
CDK6	CCAGATGGCTCTAACCTCAGT	AACTTCCACGAAAAAGAGGCTT
cyclinA	TGGAAAGCAAACAGTAAACAGCC	GGGCATCTTCACGCTCTATTT
cyclinB	TTGGGGACATTGGTAACAAAGTC	ATAGGCTCAGGCGAAAGTTTTT
cyclin D	GCTGCGAAGTGGAAACCATC	CCTCCTTCTGCACACATTTGAA
cyclinE	GCCAGCCTTGGGACAATAATG	CTTGCACGTTGAGTTTGGGT
GTSE1	CCACCGGGATGTTCTCCCT	TTCAGCCCCAACTTGTTTGGA
E2F2	CATCCAGCTCATCCGCAAGAAGG	CGGCTCCTGCACCTCCTCTG
E2F8	CCTGAGATCCGCAACAGAGAT	AGATGTCATTATTCACAGCAGGG
U6	CTCGCTTCGGCAGCACA	
miR-186-5p	GCGCAAAGAATTCTCCTTTTGGGCT	
miR-101-3p	GCGGCGTACAGTACTGTGATAACTGAA	
miR-543	ACACTCCAGCTGGGAAACATTCGCGGTGCAC	

	Sequences	
	5‘-3’	5'-3'
Si-NC	UUCUCCGAACGUGUCACGUTT	ACGUGACACGUUCGGAGAATT
Si-SNHG6-1#	GAAGGUGUAUGAAAGUCAUTT	AUGACUUUCAUACACCUUCTT
Si-SNHG6-2#	CGGCAUGUAUUGAGCAUAUTT	AUAUGCUCAAUACAUGCCGTT
mimics control	UUCUCCGAACGUGUCACGUTT	ACGUGACACGUUCGGAGAATT
miR-101-3p mimics	UACAGUACUGUGAUAACUGAA	CAGUUAUCACAGUACUGUAUU
inhibitor control	CAGUACUUUUGUGUAGUACAA	
miR-101-3p inhibitor	UUCAGUUAUCACAGUACUGUA	
